# Tracking global invasion pathways of the spongy moth (Lepidoptera: Erebidae) to the United States using stable isotopes as endogenous biomarkers

**DOI:** 10.1002/ece3.9092

**Published:** 2022-07-13

**Authors:** Nadine‐Cyra Freistetter, Gregory S. Simmons, Yunke Wu, David C. Finger, Rebecca Hood‐Nowotny

**Affiliations:** ^1^ Institute of Soil Research, Department of Forest‐ and Soil Sciences University of Natural Resources and Life Sciences Tulln Austria; ^2^ Department of Engineering Reykjavik University Reykjavík Iceland; ^3^ Finnish Meteorological Institute (FMI) Climate Systems Unit Dynamicum Kumpula Finland; ^4^ Otis Laboratory and Salinas Station, United States Department of Agriculture Animal and Plant Health Inspection Service, Science and Technology Buzzards Bay/Salinas MA/CA USA; ^5^ Sustainability Institute and Forum (SIF) Reykjavik University Reykjavík Iceland

**Keywords:** alien species, biogeochemistry, economic entomology, entomology and pathology, isoscapes

## Abstract

The spread of invasive insect species causes enormous ecological damage and economic losses worldwide. A reliable method that tracks back an invaded insect's origin would be of great use to entomologists, phytopathologists, and pest managers. The spongy moth (*Lymantria dispar*, Linnaeus 1758) is a persistent invasive pest in the Northeastern United States and periodically causes major defoliations in temperate forests. We analyzed field‐captured (Europe, Asia, United States) and laboratory‐reared *L. dispar* specimens for their natal isotopic hydrogen and nitrogen signatures imprinted in their biological tissues (δ^2^H and δ^15^N) and compared these values to the long‐term mean δ^2^H of regional precipitation (Global Network of Isotopes in Precipitation) and δ^15^N of regional plants at the capture site. We established the percentage of hydrogen–deuterium exchange for *L. dispar* tissue (P_ex_ = 8.2%) using the comparative equilibration method and two‐source mixing models, which allowed the extraction of the moth's natal δ^2^H value. We confirmed that the natal δ^2^H and δ^15^N values of our specimens are related to the environmental signatures at their geographic origins. With our regression models, we were able to isolate potentially invasive individuals and give estimations of their geographic origin. To enable the application of these methods on eggs, we established an egg‐to‐adult fraction factor for *L. dispar* (Δegg‐adult = 16.3 ± 4.3‰). Our models suggested that around 25% of the field‐captured spongy moths worldwide were not native in the investigated capture sites. East Asia was the most frequently identified location of probable origin. Furthermore, our data suggested that eggs found on cargo ships in the United States harbors in Alaska, California, and Louisiana most probably originated from Asian *L. dispar* in East Russia. These findings show that stable isotope biomarkers give a unique insight into invasive insect species pathways, and thus, can be an effective tool to monitor the spread of insect pest epidemics.

## Graphical abstract

To help slow the spread of invasive insects that cause ecological and economic burdens worldwide, we investigated a reliable method to track back an invaded insect’s natal origin. We confirmed that the natal isotopic values of spongy moths were related to the environmental signatures at their geographic origins. Through regression analysis, estimations of the geographic origin of potentially invasive individuals were possible.

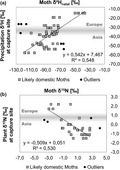

## 1 INTRODUCTION

The global economic and ecological burden posed by invasive insect species is high, with costs rising to $70 billion dollars per year (Bradshaw et al., 2016). The ability to identify the natal origin of a pest insect captured on non‐native territory would significantly support the implementation of pest management strategies. Therefore, the determination of invasive insects' natal origins has been a topic of investigation for many years, but has largely focused on genetic analysis (Picq et al., 2018; Wu et al., 2015, 2020). While genetic analyses can yield insight into a population's geographic origin, it does not provide the nuanced data often required for effective management of a newly arrived invasive species, such as whether the individual was born on site (domestic/local) or transported in (exotic). On the other hand, stable isotope analysis (SIA), particularly of hydrogen, nitrogen, sulfur, and oxygen, is a useful tool for tracking an individual's recent whereabouts (Bowen & West, 2008; Hobson, 1999).

The European spongy moth *Lymantria dispar dispar* (Lepidoptera: Erebidae, Linnaeus 1758) (subsequently referred to European *L. dispar* and formerly known as the gypsy moth) is native to all of temperate Eurasia and northern Africa, and was brought to the United States between 1868 and 1869 near Boston, Massachusetts, from where it escaped and spread shortly after (Fernald & Forbush, 1896). In the United States, there are 400 different tree species European *L. dispar* larvae feed on (US Department of Agriculture et al., 1981). Additionally, the moth faces no major natural enemies, competitors, or diseases in North America that would control populations like in their native habitat (Liebhold et al., 1995). Consequently, the moth has been able to gradually expand its distribution. The area infested by European *L. dispar* is confined to the northeastern United States and the eastern maritime provinces of Canada, with an advancing front slowly moving in a southwesterly direction (Epanchin‐Niell & Liebhold, 2015). The population periodically builds to outbreak levels that can result in serious economic, environmental, and public nuisance problems (Liebhold et al., 2000; Thorpe et al., 2006). Since 1924, tens of millions of hectares have been defoliated in the United States forests by the European *L. dispar* (Sharov et al., 2002), leaving regional economies with costly pest mitigation and prevention measures (Bigsby et al., 2014; Epanchin‐Niell & Liebhold, 2015; Jardine & Sanchirico, 2018).

The natural expansion of European *L. dispar* in North America advances relatively slowly with 13 miles/year, which primarily is a consequence of the female's flight incapability (Reineke & Zebitz, 1998). This is an advantage for pest management, as egg masses can be found very close to the pupation sites (Fernald & Forbush, 1896). Human activities near the pupation sites, however, have significantly accelerated the spread throughout the country, either through moths placing eggs on vehicles, cargo, and gear or through displacement of infested natural resources like wood (Bigsby et al., 2011; Continental Dialogue, 2019). In addition to that, the intensifying container shipping from East Asia, including eastern China, Japan, the Republic of Korea, and eastern Russia, has opened a substantial entry pathway to North America for the Asian spongy moth *Lymantria dispar asiatica* (which include *L. dispar asiatica* [Vnukovskij] and *L. dispar japonica* [Motschulsky]) (subsequently referred to as Asian *L. dispar*) (Gray, 2017; Paini et al., 2018). One major difference between the Asian and European *L. dispar* is that females of the former subspecies are capable of strong and directed flight. The brightly lit shipping ports have been shown to attract Asian *L. dispar* females that can lay eggs on vessel superstructure and shipping containers (Gray, 2017; Wallner et al., 1995). If the Asian *L. dispar* were to establish in North America, they can hybridize with European *L. dispar* and produce fertile offspring with flight or gliding capable females, which might accelerate population expansion of the pest, as some researchers believe (Gray, 2017; Keena et al., 2007; Robinet & Liebhold, 2009). The Asian *L. dispar* is also adapted to colder climates and higher altitudes and therefore has an even broader host range than European *L. dispar*, with 500 host species, including several coniferous trees (Trotter et al., 2020). The Asian *L. dispar* is not yet established in the United States, but adult Asian *L. dispar* males of unknown natal origin were captured in several US states in the past years, which gave reasons for concern for natural biospheres and called for action to intensified pest management and prevention measures (US Department of Agriculture Animal and Plant Health Inspection Service, 2019).

There are *L. dispar* surveillance and eradication programs, both inside and outside of the currently infested regions of North America (Continental Dialogue, 2019; Epanchin‐Niell & Liebhold, 2015; Liebhold et al., 2021; Sharov et al., 2002; Sharov & Liebhold, 1998). Internationally, there are strict codes of practices for the shipping industry that give legal grounds to accept only guaranteed infestation‐free ships and cargo into North American ports (Canadian Food Inspection Agency & United States Department of Agriculture, 2022; IMO/ILO/UNECE, 2014; USDA Forest Service, 2014). Despite these practices, European and Asian *L. dispar* detections are made in the United States (Bigsby et al., 2011; Paini et al., 2018). To effectively slow the spread, limit the establishment of *L. dispar* across North America and to locally eradicate new outbreaks, it will be important to determine whether newly detected moths or eggs are from a locally established population or are recent arrivals from imported cargo (Gray, 2017). In some instances, genetic analysis of a specimen may be capable of determining a linage's geographic descent (Wu, 2016; Wu et al., 2015, 2020). However, stable isotope analysis (SIA) can help control and eradication programs by adding important information on the geographic origin and introduction pathways of an individual, the insect's diet, and alternative host species, which can help clarify whether a detection is newly introduced or has been locally established (Hungate et al., 2016; International Atomic Energy Agency, 2009).

The stable isotope signatures of an individual reflect the natural, geographically dependent, variations of isotopic signals in precipitation, soil, and vegetation of the individual's origin (Cameron, 2005). If the location of the individual changes rapidly (in relation to the endogenous turnover rate of its tissue), it will retain its origin's isotopic profile while already living in the new destination (International Atomic Energy Agency, 2009). Since chitin structures like insect exoskeletons have a slow turnover rate, isotopic signals of the chitin tissue, such as the deuterium–hydrogen ratio (δ^2^H) and the ^15^N/^14^N ratio (δ^15^N), are suitable biomarkers to track an insect's recent migration (Hungate et al., 2016). Tables 1 and 2 show the mean annual δ^2^H values in precipitation and mean annual δ^15^N values in foliage depending on the geographic regions (Bowen & West, 2008; Terzer et al., 2013).

**TABLE 1:** Geographic zones depending on average annual δ^2^H in precipitation (colors and values adapted from Terzer et al., 2013 world map for easy comparison)

δ^2^H zone	Upper boundary (‰)	Lower boundary (‰)	Corresponding regions (corresponds to climate)
H1	100.0	16.1	Sahara, Saudi Arabian Desert
H2	16.1	0.1	Sahara, Saudi Arabia, East Africa, Central Australia
H3	0.1	−15.9	Central and South Africa, Australia, Pakistan, Gulf of California, Florida, Cuba, Northwestern Brazil
H4	−15.9	−31.9	Continental South America, Southern US, India, West Africa, Australian Coasts
H5	−31.9	−47.9	East‐Central US, West Europe, Central Asia, Southeast Asia, Southeast China, New Zealand, Southeast Brazil
H6	−47.9	−63.9	Central Europe, Northern US, Japan, Central America
H7	−63.9	−79.9	North Europe, Northeast US, South Russia, Southern Argentina
H8	−79.9	−95.9	Alaska, Northwest US, Southern Canada, Central Russia, Lapland, Himalaya
H9	−95.9	−111.9	Central Canada, Northern Russia, Himalaya, Andes
H10	−111.9	−127.9	
H11	−127.9	−143.9	Northern Canada, Greenland, East Siberia, Andes Summits
H12	−143.9	−159.9	
H13	−159.9	−300.0	North coast Greenland

**TABLE 2:** Geographic zones depending on average annual δ^15^N in plants (colors and values adapted from Bowen & West, 2008 world map for easy comparison)

δ^15^N zone	Upper boundary (‰)	Lower boundary (‰)	Corresponding regions (corresponds to vegetation)
N1	4.7	3.9	Sahara, Saudi Arabian desert, Pakistan
N2	3.9	3.3	West India, East Africa, Sub‐Sahara
N3	3.3	2.8	East India, Australia, Gulf of California, East Brazil
N4	2.8	2.2	South Africa, Mexico, Thailand, Caspian Sea, Cuba, Continental South America
N5	2.2	1.7	
N6	1.7	1.1	Central Africa, Central Brazil, Chile, West and South US, South Europe
N7	1.1	0.5	Central Europe, Central‐East US, East China, Kazakhstan
N8	0.5	0.0	Southern Russia, East Europe
N9	0.0	−0.5	Northern US, Scandinavia, East Russia
N10	−0.5	−1.1	Mongolia, Japan, Northern China
N11	−1.1	−1.6	Alps, Himalaya
N12	−1.6	−2.1	Alaska, Western Norway
N13	−2.1	−2.4	Northern Russia, Northern Canada, Andes
N14	−2.4	−2.7	
N15	−2.7	−3.1	Southern Canadian Archipelago, East Siberia
N16	−3.1	−3.6	
N17	−3.6	−4.3	Northern Greenland, Northern Canadian Archipelago, Andes summits
N18	−4.3	−8.5	

The *L. dispar* is exceptionally suitable for SIA to track the natal origins, as it only feeds in the larval stage (Drooz, 1985). The 40 days feeding period of larvae starts between May and July after hatching. In the short postmetamorphosis adulthood (6–10 days), moths do not possess a digestive system. They solely mate, lay the overwintering egg masses from which larvae hatch the following spring, and die (Drooz, 1985; US Department of Agriculture et al., 1981). A *L. dispar*'s isotopic composition therefore is a direct derivative of the vegetation's isotopic composition it fed on as a larva (Hungate et al., 2016), and it does not significantly change during the adult stage. As the eggs are formed inside the females' adult body, they reflect the mother's isotopic values (International Atomic Energy Agency, 2009; Montgomery, 1982) until they hatch and start feeding the next year.

A small proportion of an insect's hydrogen isotope value, however, is only loosely bound in the tissue and can be exchanged with hydrogen in the water of the ambient air (Hungate et al., 2016; Qi & Coplen, 2011). Thus, the isotopic value of tissue changes slightly (also postmortem) within a certain equilibration period, depending on the surrounding air conditions. This phenomenon is called hydrogen–deuterium exchange, and its magnitude is determined by the species‐specific percentage of exchangeable hydrogen (Hungate et al., 2016; International Atomic Energy Agency, 2009; Wassenaar & Hobson, 2003). The relevance for origin studies and the exact percentage for *L. dispar* have never been studied before.

A moth's origin is not transparent because: first, land‐based vehicles are mostly not inspected for life stages. Second, commercial maritime vessels, though rigorously inspected at North American ports, have multiple stop routes, which are not assessable for most cases. Eggs may remain undiscovered for a longer period and genetic analysis rather addresses the heritage of a strain than an individual's recent whereabouts. This study explores if SIA measurements of *L. dispar* can be a reliable method to identify the natal origins of individual life stages for any given scenario. Specifically, we asked the following questions: (1) Are the δ^2^H and δ^15^N values of spongy moths reliable markers to distinguish recent intruders from established/native populations on a global scale? (2) How accurately can we determine a moth's origin based on their δ^2^H and δ^15^N values? (3) What is the exact percentage of exchangeable hydrogen (P_ex_) for spongy moth tissue? (4) Are the same methods applicable to eggs?

Our study aims to help develop stable isotope tools for pest pathway analysis and control programs in general, as the principles used in this study can be applied to all chitin tissues of insects (Bowen et al., 2005; Bowen & West, 2008; Hobson et al., 2004; Hungate et al., 2016; International Atomic Energy Agency, 2009; Mekki et al., 2016).

## 2 MATERIALS AND METHODS

To assess a reproducible method to determine the natal provenance of possibly exotic Asian *L. dispar* or European *L. dispar* samples (as done before for other insect pests by Hungate et al., 2016), we measured field‐captured and laboratory‐reared moths and eggs for their deuterium and nitrogen isotopic signatures.

### 2.1 Sampling and measurements

*Lymantria dispar* eggs of uncertain origin were sampled from ships berthing in the United States ports in Alaska, California, Louisiana, and Orlando between 2013 and 2016 (*n* = 8–10 eggs per location).

Adult life stages were collected from England, France, Germany, Slovakia, Spain, China, Japan, Korea, Russia, and the United States (*n* = 10–12 per location) for reference. These males were caught with sticky delta traps (Scentry Biologicals, Inc.) containing *L. dispar* sex pheromone Disparlure (Scentry Biologicals, Inc.) between 1992 and 2016. All samples were dried at 60°C and stored at −20°C before shipping. Table 3 shows all the samples analyzed in this study with local features of the capture sites.

**TABLE 3:** Metadata for the capture sites of the field‐collected spongy moth samples analyzed in this study

Country	Collection site	Latitude	Longitude	Height above sea level	Local relief	Mean annual temperature	Annual precipitation	Local urbanization	Domestic *L. dispar* colonies known	Local precipitation δ^2^H	Local plant δ^15^N	Life stage	Year of collection	Samples measured
		(°N)	(°E)	(m)		(°C)	(mm)		(yes/no)	(‰)	(‰)			(#H/#N)
France	Porto‐Vecchio, Corsica	41.55	5.44	120	Mount.	14.80	646	Urban	Y	−19.20	1.3	Adult	1993	4/3
Germany	Ramstein	49.30	7.30	245	Hilly	8.00	640	Semiurban	Y	−30.50	0.5	Adult	1994	4/2
Slovakia	Banska Stiavnica	48.00	19.00	520	Hilly	9.17	557	Semiurban	Y	−37.20	0.3	Adult	2012	4/1
Spain	Alcala de los Gazules	36.00	−5.00	170	Flat	18.21	570	Urban	Y	−14.50	1.7	Adult	1994	5/2
Spain	El Castano	40.00	−4.00	500	Hilly	18.21	570	Rural		−17.00	2.2	Adult		0/2
UK	London	51.50	0.12	5	Flat	11.00	517	Urban	Y	−28.00	0.7	Adult	1995	4/2
China	Beijing, Daxing County	40.00	116.00	40	Hilly	12.10	578	Urban	Y	−34.67	0.70	Adult	1993	4/1
China	Liuan, Anhui	31.70	116.00	75	Flat	16.13	975	Urban	Y	−53.67	0.00	Adult	1995	4/2
China	Tengzhou, Shandong	35.07	117.15	63	Flat	12.00	750	Semiurban	Y	−28.00	1.10	Adult		0/3
China	Suihua, Heilongjiang	46.00	126.00	200	Flat	3.96	521	Semiurban	Y	−57.67	−0.60	Adult	2008	4/3
China	Changbaishan, Jilin	43.00	128.00	2400	Mount.	4.75	109	Rural	Y	−71.00	−0.80	Adult	2011	4/1
Japan	Honshu	35.81	139.62	70	Hilly	14.00	2000	Semiurban	Y	−48.75	−1.1	Adult		0/6
Japan	Koshunai, Hokkiado	43.20	141.5	25	Hilly	9.00	1108	Rural	Y	−38.00	−1.5	Adult	1992	4/3
Russia	Vladivostok Port	43.00	132.00	10	Hilly	4.79	799	Urban	Y	−39.75	−0.7	Adult	1995	4/3
South Korea	Seoul, Dongpae‐ri	37.70	126.70	30	Hilly	12.71	1345	Urban	Y	−47.75	−0.4	Adult	1992	4/3
South Korea	Seoul	35.81	139.62	70	Hilly	14.00	2000	Semiurban	Y	−48.75		Adult		0/3
US	St. Louis County, Minnesota	47.51	−92.00	450	Flat	4.33	786	Rural	Y	−56.80		Adult	2016	1/1
US	Carlton County, Minnesota	46.00	−92.00	325	Flat	4.33	786	Rural	Y	−46.33	−0.6	Adult (head)	2016	4/1
US	Cook County, Minnesota	48.00	−92.00	400	Flat	4.33	786	Rural	Y	−51.60	−0.6	Adult	2016	4/1
US	Lake County, Minnesota	47.70	−94.40	500	Flat	4.33	786	Rural	Y	−47.00		Adult	2016	2/0
US	Brown, Wisconsin	44.50	−88.00	180	Flat	6.79	750	Semiurban	Y	−26.50	−0.4	Adult	2016	4/1
US	Chittenden, Vermont	42.00	−73.00	305	Hilly	6.88	1027	Semiurban	Y	−25.25	−0.6	Eggs	2016	4/2
US	*Juneau, Alaska (Ship)	58.00	−134.00	10	Mount.	4.75	2341	Semiurban	N	−112.00	−4.0	Eggs	2014	3/2
US	*Long Beach, California (Ship)	33.40	−118.20	136	Flat	17.20	379	Urban	N	−16.25	1.0	Eggs	2014	2/1
US	*New Orleans, Louisiana (Ship)	30.00	−90.00	0	Flat	21.46	1333	Urban	N	−21.80	2.0	Eggs	2015	2/1
US	*Portland, Oregon (Ship)	45.50	−122.60	55	Hilly	12.46	2600	Urban	Y	−43.25	−2.0	Eggs	2013	2/2
OTIS Lab	Ctrl LDAM	41.65	−70.50	30	Flat	10.71	1112	Urban	–	(−47.00)	–	Adult	2017	10/2
OTIS Lab	*Ctrl LDAM	41.65	−70.50	30	Flat	10.71	1112	Urban	–	(−47.00)	–	Eggs	2017	6/0
OTIS Lab	Ctrl NJSS	41.65	−70.50	30	Flat	10.71	1112	Urban	–	(−47.00)	–	Adult	2017	4/3

*Note:* For the control groups (“ctrl”, Mongolian Asian *Lymantria dispar* “LDAM” and New Jersey strain of European *L. dispar* “NJSS”, last three lines), the value for “local precipitation δ^2^H” is the local tap water value that was used for the artificial diet, written inside parentheses.

The USDA‐APHIS‐PPQ‐Otis laboratory insectary reared two spongy moth control groups (“LDAM”: *Lymantria dispar asiatica*, a Mongolian strain and “NJSS”: European *L. dispar*, a New Jersey strain) from February to March 2017 (26.8 ± 1°C, relative humidity: 75 ± 5%, photoperiod: 12 h). The larvae were fed an artificial diet consisting of 12% wheat germ and 82% tap water supplied by surface water bodies (Cape Cod Commission, 2017) using standard protocols described by Bell et al. (1981) and Miller et al. (1996). During LDAM's rearing, two different packages of wheat germ were used to prepare the diet, which introduced isotopic variability to the results. (H. Nadel, USDA APHIS PPQ S&T Otis Laboratory May 2019 Pers. Comm.). The control groups served as reference for: (1) the naturally occurring isotopic variance (standard deviation) among identically raised moths, (2) the isotopic difference between moths from different genetic strains raised under the same conditions, (3) the introduction of isotopic variance from the diet, and (4) the offset between isotopic values of adult moths and their eggs.

We analyzed samples together with reference materials (RMs) for their isotopic hydrogen (δ^2^H) and nitrogen (δ^15^N) ratio at the University of Natural Resources and Life Sciences isotope laboratory (Tulln, Austria) between 2017 and 2019. For deuterium analysis, quadruplet samples of 0.2 ± 0.025 mg each of the moths' tarsus (lowest part of the leg, dominantly consisting of chitin) were weighed into silver capsules for solids (3.3 × 5 mm, IVA Analysentechnik). Eggs were sampled as a whole. A full complement of in‐house water reference materials (measured against VSMOW and VSLAP) and the international standards IAEA CH7 PEF1 (δ^2^H = −100.3‰), USGS 43 Indian hair (δ^2^H = −44.4‰), IVA Casein 139443 (δ^2^H = −113.0‰), and IVA NBS22 (δ^2^H = −116.9‰) were used. The δ^2^H measurements were conducted with a TC/EA system (Thermo Fisher Scientific), a ConFlo III open split interface (Thermo Fisher Scientific), and a Delta^PLUS^ XP isotope‐ratio mass spectrometer (IRMS, Thermo Fisher Scientific). The H_3_^+^ contribution (“H3‐factor”) was determined before every measurement.

For the δ^15^N analysis, 2 ± 0.3 mg of moth tissue were sampled into tin capsules for solids (5 × 8 mm, IVA Analysentechnik). Due to a lack of samples, multiple specimens were combined into one sample for the δ^15^N analysis. This introduced additional variance to the δ^15^N results. We used IVA Urea (δ^15^N = −0.36‰), IAEA 600 Caffeine (δ^15^N = 0.9‰), and IVA Casein 165389 (δ^15^N = 5.9‰) as reference materials. The δ^15^N was analyzed with an EA (Flash 2000) and IRMS Delta V system (Thermo Fisher Scientific).

Samples and RMs were dried in a desiccator at room temperature for at least 48 h prior to measurements. Results were conventionally reported as ratios (^2^H/^1^H or ^15^N/^14^N) in delta notation (δ^2^H or δ^15^N) in per mil (‰) deviation from the Vienna Standard Mean Ocean Water VSMOW for δ^2^H (Equation 1) and from technical air for δ^15^N (International Atomic Energy Agency, 2009) in the form δH12=H12/H11sampleH12/H11VSMOW−1 (Equation 1).

We applied the same methods for the control (Ctrl) group studies. Quadruplet samples of Ctrl‐European *L. dispar* and Ctrl‐Asian *L. dispar* were prepared and measured together for δ^2^H and δ^15^N. A second set of Ctrl‐Asian together with Ctrl‐Asian eggs were prepared and measured afterward, by another person, to define the egg‐to‐adult offset.

We used the reference materials' expected versus measured linear regression to obtain a calibration equation. We corrected the insect values for the measurement inaccuracy using the equation $x=\frac{y-d}{k}$ (Equation 2), where *y* is the measured sample value, *k* is the slope, and *d* the intercept of the calibration curve.

### 2.2 Egg‐to‐adult conversion

Eggs generally showed a high negative offset from adult moths (up to 30‰). We established an egg‐to‐adult fraction factor (16.3 ± 4.3‰) that allows to approximate the mother's isotopic value. We added the offset to eggs' measured values after the correction of the measurement inaccuracy (see above) and analyzed them like adult moth samples from thereon.

### 2.3 Hydrogen–deuterium comparative equilibration experiment

In order to obtain the fixed (nonexchangeable) value $\delta 2H_{\text{natal}}$, we determined the percentage of exchangeable hydrogen ($P_{\mathrm{ex}}=1-P_{\mathrm{nex}}$) in comparative equilibration experiments (Hungate et al., 2016; Wassenaar & Hobson, 2003) and removed the exchangeable part ($\delta 2H_{\mathrm{Amb}})$ from the moth value. Two groups of five 0.2 ± 0.025 mg samples from Wisconsin moths' tarsus were separately equilibrated with two different microatmospheres for 1 week ($\delta 2H_{\text{Group}1}=+4.4‰$ and $\delta 2H_{\text{Group}2}=-157‰$) until the equilibrium $\delta 2H_{\text{sample}}=P_{\mathrm{ex}}*\delta 2H_{\mathrm{Amb}}+P_{\mathrm{nex}}*\delta 2H_{\text{natal}}$ (Equation 3) was stable. Then the samples were dried in a vacuum oven at 60°C for 4 days to remove moisture and transferred to measurement immediately thereafter (Qi & Coplen, 2011). Analytes were not exposed to ambient air in the laboratory for longer than 2 h before measurements. With the two distinct equilibrated groups, we calculated the $P_{\mathrm{ex}}=\frac{\delta 2H_{\text{Group}2}-\delta 2H_{\text{Group}1}}{\delta 2H_{\mathrm{Amb}2}-\delta 2H_{\mathrm{Amb}1}}\;$ (Equation 4). With knowledge of the $P_{\mathrm{ex}}$ and the identity $P_{\mathrm{nex}}=1-P_{\mathrm{ex}}$, we calculated the natal signature $\delta 2H_{\text{natal}}=\frac{\delta 2H_{\text{Group}}-P_{\mathrm{ex}}*\delta 2H_{\mathrm{Amb}}}{1-P_{\mathrm{ex}}}\;$ (Equation 5) (International Atomic Energy Agency, 2009).

### 2.4 Expected insect values

We approximated the expected chitin δ^2^H values by inserting the capture sites' OIPC values into the regression by Hungate et al. (2016) for Japanese beetles (*Popillia japonica*). For the expected δ^15^N, we used a model for the annual average δ^15^N composition of plants worldwide (Bowen & West, 2008).

### 2.5 Outliers

Values with an offset greater than 20‰ (δ^2^H) or 2‰ (δ^15^N) between the individual natal moth value and the expected insect value (see above) were considered outliers. In addition, we considered values δ^15^N > 5‰ as outliers, according to the natural boundary in Bowen and West (2008). Outliers were automatically submitted to origin backtracking.

### 2.6 Regression analysis

Based on trap location details and satellite images, we estimated the geographical coordinates of the moths' capture sites. These data were not recorded upon capture, but needed for the capture site's isotopic signal estimates. We retrieved the monthly averages of δ^2^H in precipitation at the moth's capture sites during the assumed feeding period from the Online Isotopes in Precipitation Calculator (OIPC) (Bowen, 2018; Bowen et al., 2005; IAEA and WMO, 2015; Welker, 2000). We corrected this expected δ^2^H insect values for climate change if moths were caught before 2000 (Liu et al., 2018). An evaluation for land‐use change in the region would have been useful but was out of scope for this study.

Subsequently, we performed a correlation analysis between all individuals that had passed the outlier tests or were confirmed domestic (specimens from Minnesota, US) and the expected environmental signature at their collection site. Isolated values with a disproportionately big negative impact on the correlation were removed manually (seven samples for δ^2^H and four samples for δ^15^N).

All moth values were regressed into their corresponding δ^2^H_precipitation_ and δ^15^N_plant_ values by the obtained regression model. The goodness of fit was evaluated for the residuals between the regressed and the environmental values (Δ_modeled‐precipitation_ δ^2^H and Δ_model‐plant_ δ^15^N) in a two‐dimensional plot.

### 2.7 Origin analyses

To detect “likely exotic” colonies (as opposed to “likely domestic”), we performed a heteroscedastic two‐tailed Student's *t* test at a 0.05 significance level (0.01 level for δ^2^H of field‐collected adult moths due to high sensitivity) between the moths' isotopic values and the expected insect values. Outliers and values that failed the *t* test were matched with their corresponding δ^2^H or δ^15^N geographical zone (see Tables 1 and 2).

Additionally, USDA‐intern genetic analysis of some samples (methods not shown here) (Bogdanowicz et al., 1993; Garner & Slavicek, 1996; Wu et al., 2020) gave external validation of some of the model‐given predictions of origins.

## 3 RESULTS

### 3.1 Natural isotopic variation and egg‐to‐adult conversion

An Asian *L. dispar* and a European *L. dispar* strain were reared at the USDA OTIS Laboratory's insectary and measured for their δ^2^H and δ^15^N values (Table 4). The average isotopic variation for moths raised under identical conditions (average distance between minimum and maximum) was 11.9‰ for δ^2^H and 1.3‰ for δ^15^N. The average egg‐to‐adult offset for spongy moths was Δ_egg_ δ^2^H = 16.30 ± 4.3‰ (not assessed for δ^15^N).

**TABLE 4:** Measurement results for the natal δ^2^H and δ^15^N of Asian and European *Lymantria dispar* control (Ctrl) strains (adults/*eggs) reared under identical conditions in the USDA insectary

Sample	Expected insect δ^2^H^a^ (‰)	δ^2^H			δ^15^N		
		Mean (‰)	SD (‰)	Min/max (‰)	Mean (‰)	SD (‰)	Min/max (‰)
Ctrl‐European^b^	−119.43	−87.14	5.60	−95.23/−83.25	5.84	0.43	5.58/6.33
Ctrl‐Asian‐1^b^	−119.43	−101.59	5.67	−107.81/−96.65	3.74	1.25	2.85/4.62
Ctrl‐Asian‐2^c^	−119.43	−134.68	4.74	−141.95/−128.96	–	–	–
*Ctrl‐Asian‐2^c^	−119.43	−146.08	4.68	−137.52/−125.96	–	–	–

*Note*: Adults of measurement group Ctrl‐Asian‐2 and eggs of measurement group *Ctrl‐Asian‐2 might have not been completely dry.

^a^ Derived from the average local tap water value used to prepare the artificial diet.

^b^ Prepared and measured together.

^c^ Prepared and measured together afterward by a different person.

The offset between the Ctrl‐European and Ctrl‐Asian‐1 medians was 15.9‰ for δ^2^H and 1.88‰ for δ^15^N, thus almost as low as the average natural variation for *L. dispar*. The maximum distance between Ctrl‐European and a Ctrl‐Asian was as high as 24.6‰ for δ^2^H and 3.5‰ for δ^15^N.

### 3.2 Hydrogen–deuterium exchange correction

The percentage of exchangeable hydrogen (P_ex_) for spongy moths was 8.2% (Table 5). All obtained P_ex_ values were in between the plausibility thresholds of 6% < P_ex_ < 14% (Qi & Coplen, 2011). The maximum observable isotopic shift of a *L. dispar* due to P_ex_ correction was Δδ^2^H = ±1.67‰. The reference material Casein 139443 was the most comparable RM to spongy moth tissue in terms of P_ex_.

**TABLE 5:** Percentage exchangeable hydrogen (P_ex_) for insect samples and reference materials (RMs) obtained from comparative equilibration experiments

Insect sample or RM	P_ex_ ^a^
Spongy moth (*Lymantria dispar*, Wisconsin)	8.2 ± 0.4%
Japanese beetle (*Popillia japonica*, Alabama)	10.0 ± 4.2%
RM: USGS 43 Indian Hair (−44.4‰)	6.4 ± 2.7%
RM: Casein 139443 (−113.0‰)	9.0 ± 2.9%
RM: NBS 22 (−116.9‰) (expected P_ex_: 0%)	1.7 ± 1.9%

^a^ All P_ex_ values were below the reject threshold of 14% (Qi & Coplen, 2011) and were used to correct the tissue's hydrogen–deuterium exchange with ambient air (Equation 4).

### 3.3 Outliers

The field‐collected moths' isotope values mostly clustered with the same isotopic variation as observed in the control studies (not presented here). We detected δ^2^H outliers in Corsica (France, *n* = 1), Banska Stiavnica (Slovakia, *n* = 1), Vladivostok (Russia, *n* = 1), Liuan, Anhui (China, *n* = 2), and Chittenden, Vermont (US, *n* = 2). Additionally, all moths tested from Brown County, Wisconsin (US, *n* = 4) were far outside the expected range for both isotopes.

Moths' δ^15^N in Beijing (China, *n* = 1), Tengzhou (China, *n* = 1), Suihua Heiljongjiang (China, *n* = 3), and Vladivostok port (Russia, *n* = 3) exceeded the natural limits (Bowen & West, 2008) and could suggest anthropogenic (e.g., agricultural) nitrogen input in these areas. Additionally, the striking δ^2^H outlier from Corsica (France, *n* = 1) also exceeded the natural δ^15^N limits.

In Carlton County (Minnesota, US), London (U.K.), and Koshunai (Japan), the moths' δ^2^H separated into two pairs that could be from neighboring populations (not presented here). Only one pair from Minnesota was removed as outliers.

### 3.4 Locally characteristic moth values

**FIGURE 1:**
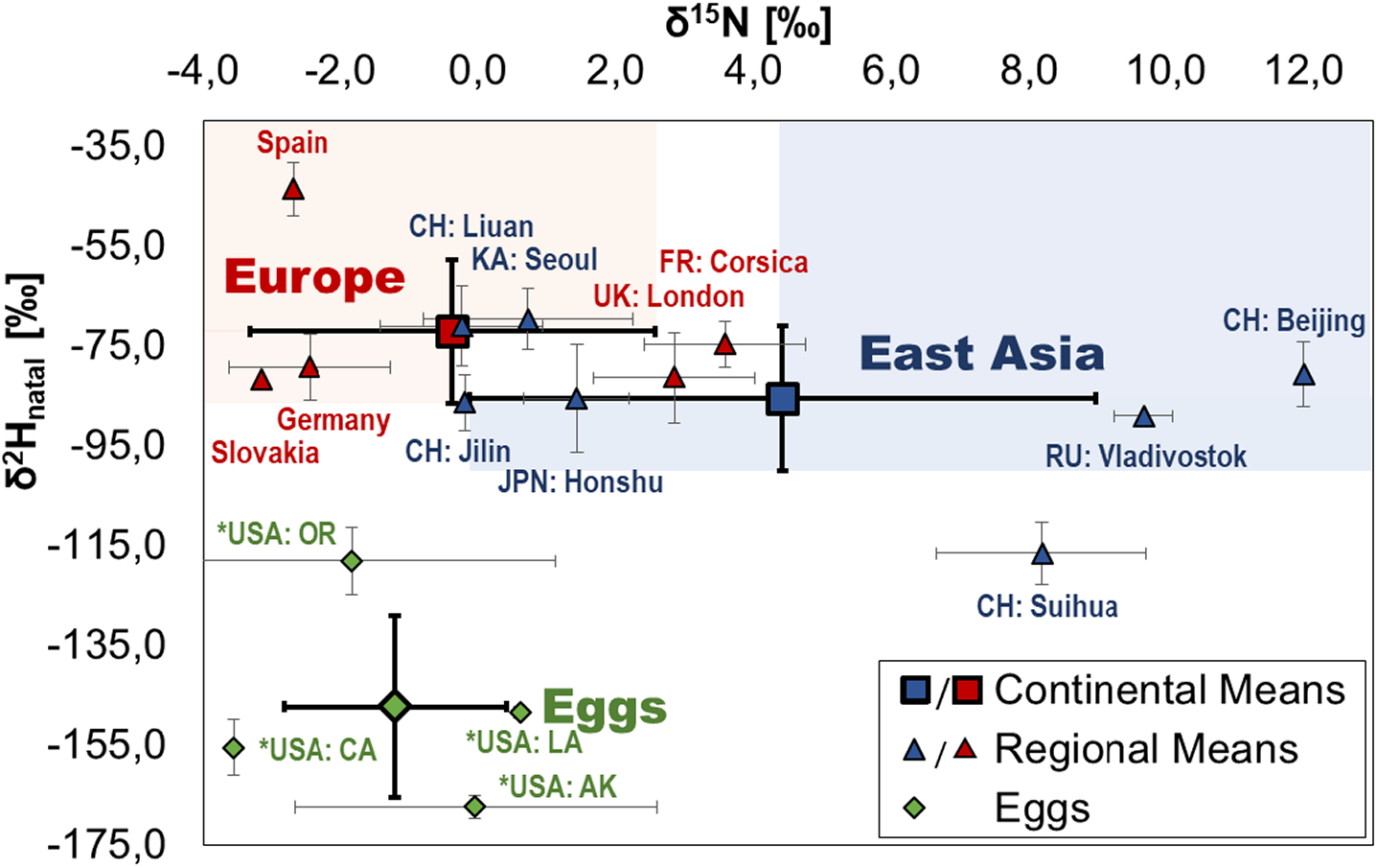
Isotopic signatures of moths (squares and triangles) and *eggs (diamonds) with standard deviations. The rectangles are continental means and the triangles regional ones. Blue stands for moths found in Asia and red for moths found in Europe. Small green diamonds are the regional means and the large green diamond is the mean of all egg samples

We obtained the natal δ^2^H and δ^15^N values by removing the percentage of exchangeable hydrogen. Generally, East Asian moths showed more δ^2^H‐negative, but more δ^15^N‐positive values than European moths (Figure 1). However, there was a variegated overlap zone between European and Asian moths. The eggs found in US harbors resembled European values in their δ^15^N, but were much more δ^2^H negative, as already reported through the egg‐to‐adult offset.

**FIGURE 2:**
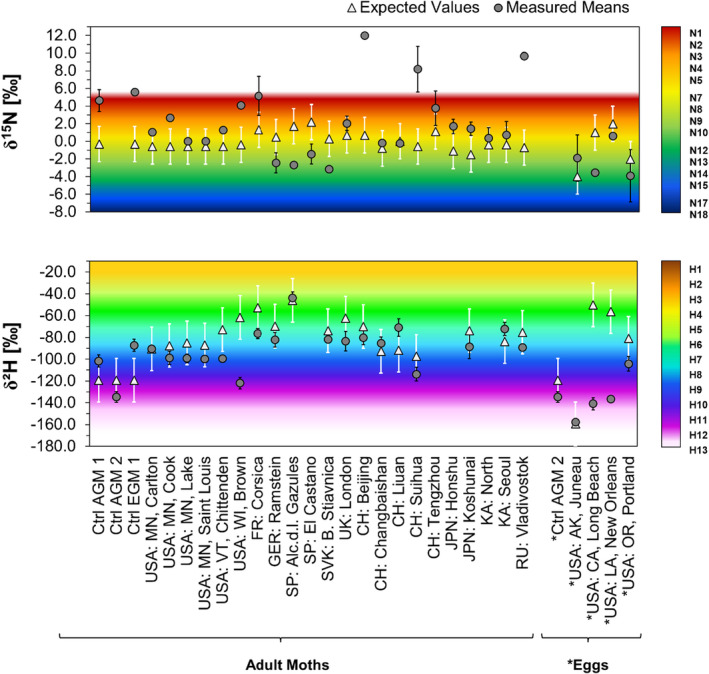
Comparison of expected insect values (white triangles) with mean δ^15^N (top row) of spongy moth adults/*eggs (gray circles) and mean δ^2^H (bottom row) of spongy moth adults/*eggs (gray circles). The black error bars show the standard deviation of moth values. The white bars show ±20‰ tolerance from the expected insect value. The color gradients refer to geographic regions for δ^15^N in vegetation (top row) (Bowen & West, 2008) and for δ^2^H in precipitation (bottom row) (Terzer et al., 2013). The expected value was retrieved from the Online Isotopes in Precipitation Calculator (Bowen, 2018)

Most moth colonies were satisfactorily close to their expected values, with 85% of δ^2^H values being categorized as “likely domestic” (Figure 2). The δ^15^N values overlapped with the expected ones only in 52% of locations, which was expected due to the uncertainties discussed in Methods. The control groups did not overlap with the expected range, which was acceptable since the moths were not fed local vegetation.

With the applied egg‐to‐adult offset for δ^2^H, the eggs from the control group overlapped identically with the expected value (Figure 2). Accordingly, this suggests that the eggs found in Juneau (Alaska) and Portland (Oregon) may have originated from a local reproducing population or from a region characterized by a similar climate and topography. For the eggs found in New Orleans (Louisiana) and Long Beach (California), the isotope data strongly suggested a geographically different origin. The δ^15^N measurements did not agree on the eggs from New Orleans (LA).

### 3.5 Worldwide moth‐origin regression

**FIGURE 3:**
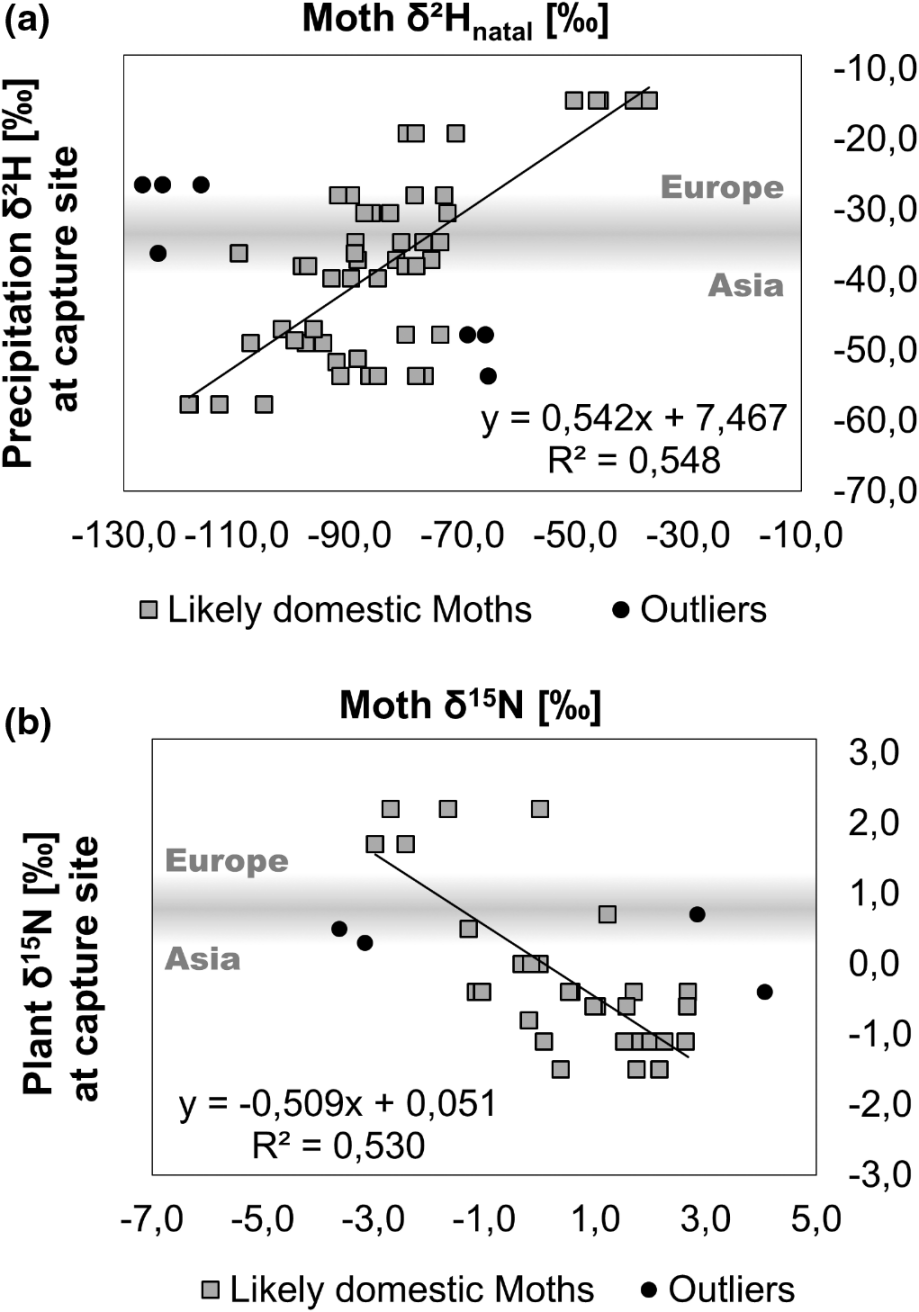
Correlation between (a) δ^2^H_natal_ of adult spongy moths and the δ^2^H_precipitation_ at their origin during the feeding period and (b) the δ^15^N of adult spongy moths and the average annual δ^15^N_plant_ at their origin. Gray squares are likely domestic moths and black circles are outliers (likely exotic)

The worldwide regression analyses yielded a moderate association between δ^2^H_moth_ and δ^2^H_precipitation_ (*R* ^2^ = .548), as well as δ^15^N_moth_ and δ^15^N_plant_ (*R* ^2^ = .530) (Figure 3), after some moths were manually removed (see Methods).

The natal environment's isotopic values plotted against the moth's values could be divided into a European and Asian section with a narrow intermixture zone between −30‰ and − 40‰ for δ^2^H and 0‰ and 1‰ for δ^15^N (Figure 3). Most US sites resembled the East Asian precipitation signatures, except for Wisconsin.

**FIGURE 4:**
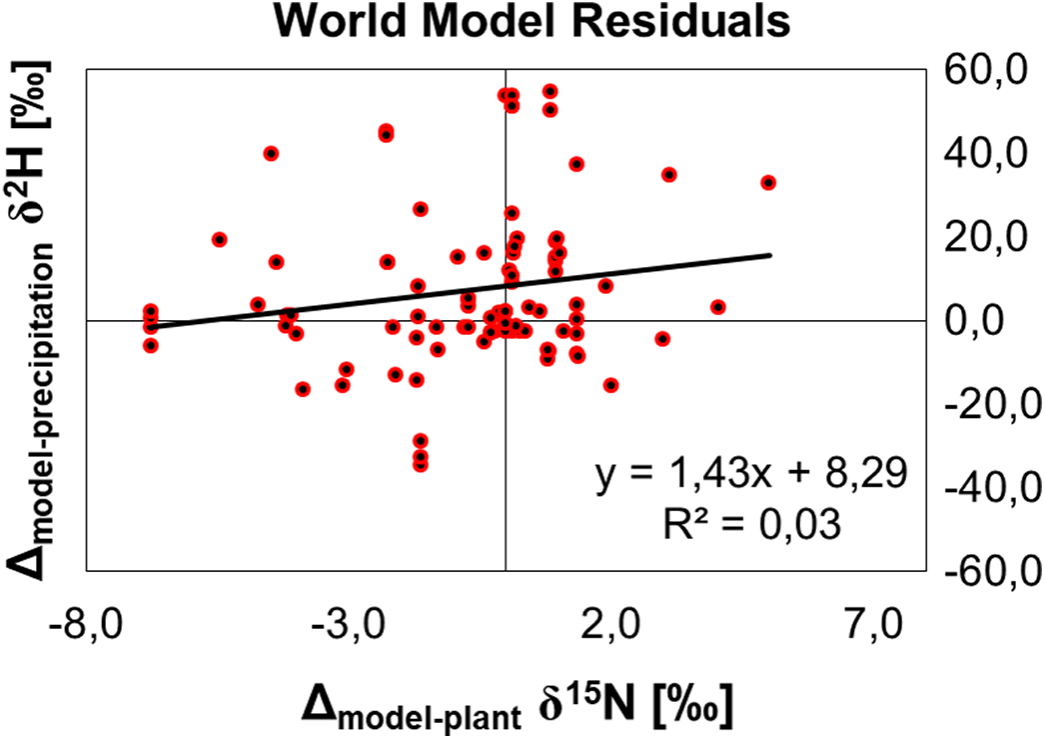
Goodness of fit for the δ^2^H_natal_ and δ^15^N worldwide models

We calculated the offset between the regressed moth values (by means of the regressions in Figure 3) and the environmental signatures Δ_modeled‐precipitation_ δ^2^H and Δ_modeled‐plant_ δ^15^N. The combined goodness of fit was moderately good (Figure 4). Most residuals were distributed in the ranges between −30‰ < δ^2^H_natal_ < 30‰ and −5‰ < δ^15^N < 5‰. We assumed no systematic error in the model, since the residuals showed a random distribution.

### 3.6 Origin analysis

**FIGURE 5:**
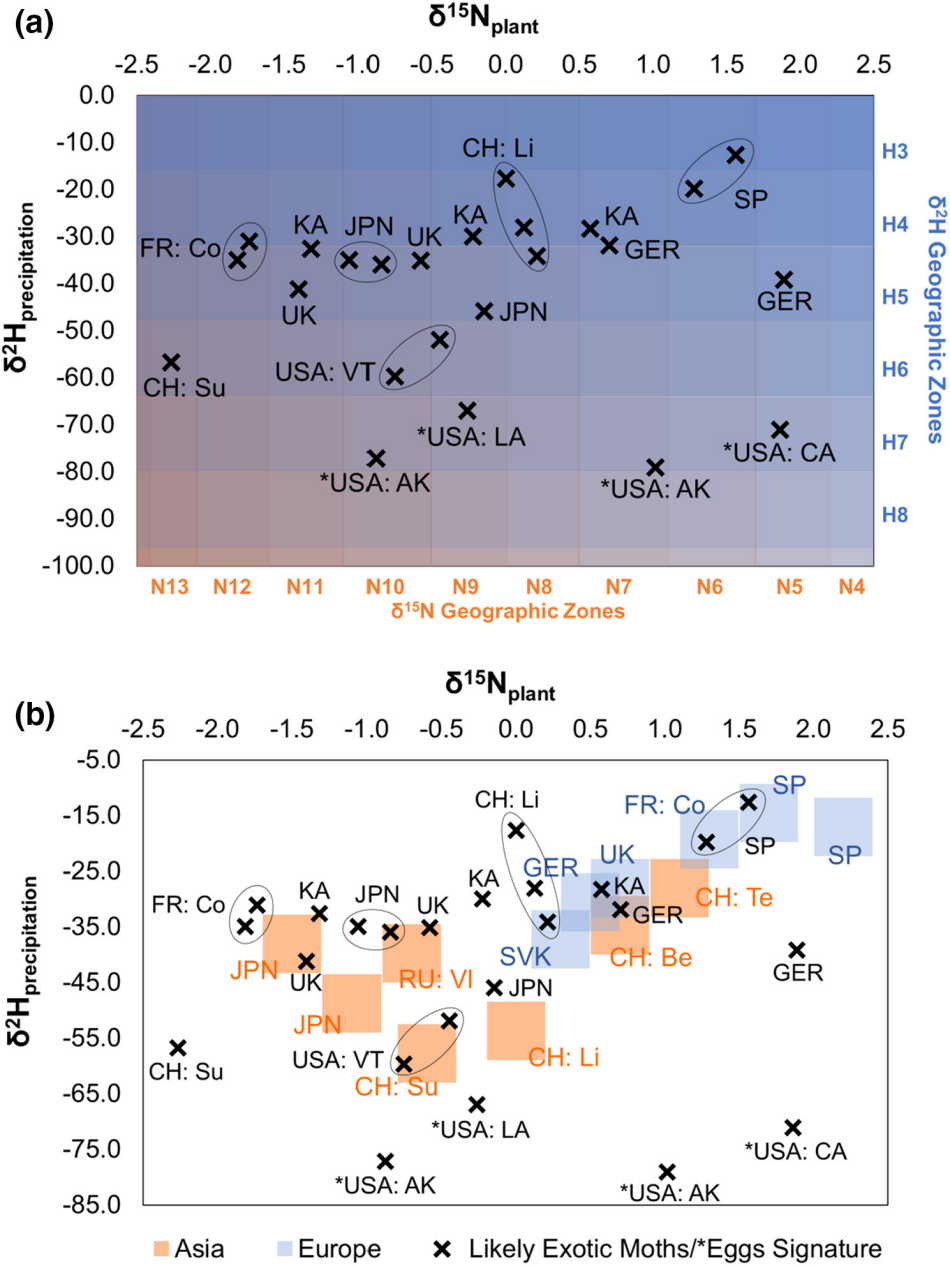
Regressed values of outliers and potentially imported moths/*eggs on the background of environmental δ^15^N–δ^2^H signatures (plant vs. precipitation). The top right corner collects hot and dry regions

Values that were earlier categorized as outliers, manually sorted out from the regression analysis, or that were classified as “likely exotic” by the Student's *t* test were regressed into their environmental δ^2^H_precipitation_ and δ^15^N_plant_ values and matched to the corresponding geographical zones (Table 6 and Figure 5).

**TABLE 6:** Regressed values of outliers and “likely exotic” moths and *eggs matched with the corresponding geographic isotopic zones (see Tables 1 and 2)

	Regressed δ^2^H	δ^2^H zone	Regressed δ^15^N	δ^15^N zone	Genetic analysis
Sample (adults)					
CH: Beijing	–	–	−6.072	18	Asian *L. dispar*
CH: Suihua	–	–	−4.786	18	Asian *L. dispar*
CH: Suihua	–	–	−5.319	18	–
CH: Tengzhou	–	–	−3.274	16	Asian *L. dispar*
CH: Liuan	−16.7	4	–	–	Asian *L. dispar*
CH: Liuan	−17.4	4	–	–	–
CH: Changbaishan	−40.0	5	–	–	Asian *L. dispar*
CH: Changbaishan	−39.2	5	–	–	–
CH: Changbaishan	−35.2	5	–	–	–
CH: Changbaishan	−43.0	5	–	–	–
KA: Seoul	−32.8	5	–	–	Asian *L. dispar*
KA: Seoul	−29.9	4	–	–	–
KA: Seoul	−28.0	4	–	–	–
KA: Seoul	−36.3	5	–	–	–
RU: Vladivostok	−52.5	6	−5.189	18	Asian *L. dispar*
RU: Vladivostok	–	–	−4.685	18	–
RU: Vladivostok	–	–	−4.794	18	–
FR: Corsica	–	–	−1.741	12	European *L. dispar*
FR: Corsica	−53.7	6	−4.169	17	–
FR: Corsica	–	–	−1.820	12	–
SVK: Banska Stiavnica	−54.3	6	–	–	European *L. dispar*
US: MN, Carlton	−29.7	4	–	–	European *L. dispar*
US: MN, Carlton	−28.0	4	–	–	European *L. dispar*
US: VT, Chittenden	−59.6	6	−0.753	10	European *L. dispar*
US: VT, Chittenden	–	–	−0.449	9	–
US: VT, Chittenden	–	–	–	–	–
US: VT, Chittenden	−63.0	6	–	–	–
US: WI, Brown	−63.5	6	–	–	–
US: WI, Brown	−70.3	7	–	–	–
US: WI, Brown	−61.4	6	–	–	–
US: WI, Brown	−57.4	6	–	–	–
Sample (eggs)					
*US: AK, Juneau	−82.7	8	–	–	Asian *L. dispar*
*US: AK, Juneau	−80.7	8	–	–	Asian *L. dispar*
*US: CA, Long Beach	−74.1	7	–	–	Asian *L. dispar*
*US: CA, Long Beach	−69.5	7	–	–	Asian *L. dispar*
*US: LA, New Orleans	−69.7	7	–	–	Asian *L. dispar*
*US: LA, New Orleans	−68.7	7	–	–	Asian *L. dispar*
*US: OR, Portland	–	–	–	–	Asian *L. dispar*
*US: OR, Portland	–	–	–	–	Asian *L. dispar*

*Note*: Genetic analysis had been carried out independently from this study (Wu et al., 2020).

The hydrogen and nitrogen analyses did not agree on outliers in most cases. Some samples from the United States (Chittenden and Brown) did not match the local expected values and were considered “likely exotic” by the model, though they were collected from local populations there. Their matching geographic zones (Tables 1 and 2), however, fit to their collection sites in the end. The eggs found on a ship in Alaska seemed to fit the local expected values perfectly at first, however, the δ^2^H *t* test classified the regressed values as “likely exotic” later. The combination of genetic analysis (Wu et al., 2020) and isotopic results suggested northeastern Russia as a possible origin. The eggs found on ships in Long Beach (CA) and New Orleans (LA) may originate from southern Russia as well. The eggs from a ship berthing in Portland (OR) were classified as “likely domestic” by both isotopes. Since there is no known established population in Oregon, and the genetic analysis identified an Asian *L. dispar* descent, this could suggest that we lack isotopic data from an Asian location that has very similar features to Oregon. Alternatively, there might be an establishing Asian *L. dispar* colony in Oregon, even though this hypothesis is very unlikely given diligent *L. dispar* survey work performed in this state. Finally, the outlier found in Corsica seemed to match East Asian environmental signatures like Japan's. This sample had not been genetically analyzed.

## 4 DISCUSSION

In this study, we analyzed field‐collected spongy moths for their natal δ^2^H and δ^15^N imprinted in their biological tissues and compared these values to the long‐term mean δ^2^H of precipitation and δ^15^N of plants at the moths'/eggs' capture sites to determine if individuals were domestic or exotic.

Our work suggests the isotopic values of *L. dispar* can be used as biomarkers of individual intruders that come from a topographically, climatic or vegetational distinct region, but are not precise enough in determining the exact origin without accompanying data like previous ports of call or genetic analysis, as demonstrated by Holder et al. (2015), where the potential ports of origin were known. For example, moths from northern United States and northeastern Asia may not be distinguishable solely by their hydrogen and nitrogen isotope ratios, as long as there are no major topographical differences between the natal origin and invasion point (e.g., mountains or a much cooler climate) (Holder et al., 2020). Moths from Minnesota, for example, matched local US as well as East Asian signatures. Isotopic analysis of hydrogen and nitrogen alone cannot guarantee that a moth/egg that resembled the expected value is in fact native at the investigated site, but it may be originating from any other place in the world that has very similar climatic, topographical, and vegetation features. Here, heavier isotope ratios, such as sulfur or strontium, may be useful in determining the true origin of the specimens, yielding information on the natal distance from the sea and underlying geology, respectively, however with increasing complexity and cost (Holder et al., 2014; Schmidt et al., 2005). This highlights that isotope studies are better at answering questions such as “Is the moth from here?” rather than open questions like “Where is the moth from?”

Generally, the δ^2^H values were significantly more sensitive, and more reliable, than δ^15^N for determining the origin of *L. dispar* (Montgomery, 1982), also because better research tools are available (Bowen, 2018; IAEA and WMO, 2015). In some of the collection sites (Carlton [Minnesota], London [UK], and Koshunai [Japan]), the measured *L. dispar* δ^2^H values split into two distinctive groupings, both of which were still near enough the expected values to be classified as “likely local.” This phenomenon could be caused by neighboring populations, one of which could be an “uphill” and the other a “downhill” population. An altitude difference of 400 m can already make up a 5‰ difference in δ^2^H values (Streifel et al., 2017). Alternatively, the phenomenon can be caused by a different primary water source of the vegetation than precipitation, that is, snow or underlying groundwater, which do not necessarily reflect the precipitation's signature (Finger et al., 2013). In the case of Minnesota, for example (one grouping at −90.4‰, the other at −67.3‰), the underlying water table has a signature similar to the close‐by Lake Superior (δ^2^H = −68‰), which is significantly lower compared to the OIPC precipitation values (δ^2^H = −46.3‰) (Foley et al., 2014). The difference of one group of larvae feeding on vegetation that primarily takes up precipitation water and another group feeding on vegetation that takes underground water provided by a near‐by surface water body would likely be detectable in *L. dispar* isotopic values (Montgomery, 1982). This not only demonstrates the high sensitivity of δ^2^H values, but also shows that any deviation from the expected value has to be assessed individually with attention to the vegetation's water sources.

Multi‐isotope comparison of regressed moth/egg values with local environmental values has the potential to yielding more clear‐cut estimates of origins, but needs more precise isotopic regression models as input on one hand, and more reference locations on the other. We concede that our model's accuracy could be improved by implementing a correction for the different heights above sea level, a parameter that should be recorded during moth collection. The discrepancy that genetic analyses classified all of the eggs to be Asian *L. dispar*, but the eggs' δ^15^N values resemble European *L. dispar* suggests that an eggs‐to‐moth conversion would likely be needed for δ^15^N values as well. A lack of samples did not allow us to conduct further studies in this direction. Neither were we able to verify our models with other plant or soil isotopic composition models, because all open‐access models were based on the same OIPC values and did not allow for independent verification. Furthermore, it must be taken into account that extreme climate events (droughts, unseasonal precipitation, flooding, etc.) or anthropogenic input (pollutants, fertilizers, etc.) might skew the actual expected value for a certain time and place (Jordan et al., 2019). The OIPC cannot address such eventualities as it is based on year on year average data (Bowen, 2018). Regional plant samples taken at the same time as the spongy moths could help clarify this skewness. To test and further develop the model, new independent samples should be tested and a catalog of local characteristic δ^2^H–δ^15^N compositions (and possibly other isotopes) of risk areas (distributing as well as receiving *L. dispar*) should be implemented, but this preliminary study demonstrates the utility of the method in a pest management context.

Another way for improved determination of the origin of *L. dispar* would be a combined analysis of stable isotopes and genetic markers. For the egg masses of unknown origin found on ships in the United States harbors (Juneau, AL; Long Beach, CA; New Orleans, LA; and Portland, OR), the genetic analysis concluded an Asian *L. dispar* origin. The eggs found in Juneau had the most negative δ^2^H of all samples measured, which points, together with the genetic analysis, toward an origin from an admixture zone that encompasses northeastern China (east of the Changbai Mountains), South Korea, and the Russian Far East (Wu et al., 2020). The highly depleted signature indicates that the species is capable of thriving in extremely snowy climates (Limbu et al., 2017), suggesting that hatchlings of this egg mass may have survived Alaska's climate.

In conclusion, our results were able to detect oversea origins of *L. dispar* egg masses that entered into the United States on ships and cargo and also demonstrated shorter range movement of *L. dispar* from ships in European ports. We identified Northeastern Asia as the most frequent location of probable origin for *L. dispar* egg masses collected from ships in ports at Juneau (AK), Long Beach (CA), New Orleans (LA), and Chittenden (VT). The responsibility of European international traffic hubs, however, should not be neglected either, as we found a strong indication of an exotic specimen from East Asia caught in Corsica in 1992, which poses a risk of being a suitable breeding place and distribution source for flight‐capable Asian–European *L. dispar* hybrids. This implies that international trade and transport play a significant role in the disseminating of *L. dispar* to the United States, and underlines the importance of current surveillance and tracking the invasion pathways for *L. dispar*. Finally, we suggest that the methods presented in this article could be applied to any insect species with a chitin‐dominant body part of low turnover rate, and similar methods for avian feather and mammal hair have been applied in previous animal migration studies (Bowen et al., 2005; Bowen & West, 2008; Foley et al., 2014; Hobson, 1999; Holder et al., 2014, 2020; Holder et al., 2015; Hungate et al., 2016; International Atomic Energy Agency, 2009; Mekki et al., 2016).

Based on the presented results, we recommend that insect trapping and surveillance, as well as control, mitigation, and inspection regimes at embarkation ports are of utmost importance to prevent uncontrollable, costly invasion. For using SIA in pest management, those factors should be considered: (1) Collection of the exact geographic coordinates of the insect capture sites, including height above sea level and where the sample was collected from (ship, cargo, plant, or other feature in the natural environment), and collection of regional vegetation samples from possible host species on the same day to track the regional δ^15^N_plant_ background. (2) Correction of hydrogen isotopic measurement results for hydrogen–deuterium exchange with the insect species‐specific percentage exchangeable hydrogen (Equation 4). (3) Conversion of eggs with an insect species‐specific egg‐to‐adult offset to backtrack the eggs' mother's isotopic signature. (4) Checking of the vegetation samples for significant skewness and applying offset to affected isotopic measurements, if necessary. (5) Regression of the insect values into their corresponding regional environmental signatures (precipitation, plants, etc.) by means of an insect‐specific regression model and comparing them to the actual environmental signatures. A combination of at least two independent isotopes should be used. (6) For a reasonable prediction of origins, native habitats, possible import pathways (e.g., through trading routes), and if accessible, genetic analyses should be taken into account.

## AUTHOR CONTRIBUTIONS

**Nadine‐Cyra Freistetter:** Data curation (lead); formal analysis (lead); investigation (lead); methodology (lead); visualization (lead); writing – original draft (lead); writing – review and editing (lead). **Yunke Wu:** Data curation (supporting); resources (supporting); validation (lead); writing – review and editing (equal). **Gregory S. Simmons:** Conceptualization (supporting); supervision (supporting); writing – review and editing (equal). **David Christian Finger:** Supervision (supporting); validation (supporting); writing – review and editing (equal). **Rebecca Nowotny‐Hood:** Conceptualization (lead); data curation (equal); formal analysis (equal); funding acquisition (lead); investigation (supporting); methodology (lead); project administration (lead); resources (lead); supervision (lead); validation (equal); writing – original draft (supporting); writing – review and editing (equal).

## CONFLICT OF INTEREST

The authors have no conflicts of interest to disclose.

## Data Availability

The moth and eggs metadata, measurement results and data analysis can be found under the DOI https://doi.org/10.5061/dryad.15dv41p0w.
